# A comparison between arthroscopic and open surgery for treatment outcomes of chronic lateral ankle instability accompanied by osteochondral lesions of the talus

**DOI:** 10.1186/s13018-020-01628-3

**Published:** 2020-03-20

**Authors:** Can Xu, Mingqing Li, Chenggong Wang, Hua Liu

**Affiliations:** Department of Orthopaedics, Xiangya Hospital, Central South University, No. 87, Xiangya Road, Changsha, 410008 China

**Keywords:** Osteochondral lesion of talus, Lateral ankle instability, Arthroscopic

## Abstract

**Background:**

This study aimed to examine the efficacy and safety of the arthroscopic treatment of osteochondral lesion of talus (OLT) and lateral ankle instability. It was hypothesized that the outcome of all-arthroscopic surgery was no worse than that of the combined open and arthroscopic surgery for treating chronic lateral ankle instability accompanied by OLT.

**Methods:**

The patients diagnosed of chronic lateral ankle instability accompanied by OLT, who were surgically treated between May 2015 and May2017, were targeted for inclusion. Specifically, patients who received the arthroscopic treatment of OLT and lateral ankle instability were enrolled in the all-arthroscopic group, while patients who received the arthroscopic treatment of OLT and open lateral ankle stabilization were enrolled in the combined open and arthroscopic group. All the patients were followed up in terms of the Karlsson Ankle Functional Score, visual analog scale (VAS) score, Tegner activity score, and American Orthopaedic Foot & Ankle Society (AOFAS) score. Meanwhile, the satisfaction and complication rates were evaluated and compared.

**Results:**

This retrospective study included a total of 67 patients, including 32 patients in the all-arthroscopic group and 35 patients in the combined group. At a minimum of 24-month follow-up, the functional outcomes were significantly improved in both groups in relation to the preoperative condition. However, the two groups did not differ significantly from each other in terms of the Karlsson score (83.1 ± 8.2 vs 81.7 ± 9.1; *P* = 0.89), the VAS score (1.8 ± 1.6 vs 2.1 ± 1.7; *P* = 0.73), the Tegner score (5.5 ± 2.3 vs 5.0 ± 2.1; *P* = 0.72), and the AOFAS score (87.7 ± 7.6 vs 86.9 ± 7.3; *P* = 0.77). In addition, the satisfaction and complication rates exhibited no significant differences between the two groups.

**Conclusion:**

In comparison with the open lateral ankle stabilization and arthroscopic treatment of OLT, the all-arthroscopic procedure showed no difference in clinical outcomes at a minimum of 24-month follow-up. Despite the benefits of minimally invasive arthroscopic procedure combined with a relatively aggressive postoperative rehabilitation protocol, the clinical outcomes for patients with chronic lateral ankle instability accompanied by OLT did not yield significant improvement.

**Trial registration:**

The present study was carried out with the approval issued by the Institutional Review Board of Xiangya Hospital (no. 202002010).

## Background

Osteochondral lesions of the talus (OLT) are the commonly seen injuries that are often resulted from acute ankle sprains or chronic ankle instability. It has been reported that up to 16%-54% of patients diagnosed of chronic lateral ankle instability are suffering from OLT [1–5]. Considering that prolonged instability of the ankle is a potential contributing factor to the development of osteochondral lesions, which can ultimately result in osteoarthritis [6], the concomitant treatment of OLT and lateral ankle instability has been indicated [7–9].

Generally speaking, the bone marrow stimulation via microfracture or retrograde drilling is usually administered for small primary OLTs [10, 11], while the osteochondral grafting treatment such as the osteochondral autograft transfer system (OATS) is adopted for large OLTs [7]. In recent years, cell-based repair techniques including autologous chondrocyte implantation have also been used [12]. Bone marrow stimulation like microfracture can be performed under arthroscope. As a minimally invasive technique and a preferred treatment for OLT, it provides fibrocartilage infill to the defect site. For patients of OLT accompanied by lateral ankle instability, previous studies suggested that the concurrent arthroscopic treatment of OLT and open lateral ankle stabilization was a reliable procedure [13, 14]. However in this procedure, an additional curved 4-cm incision was required for open lateral ankle stabilization after the arthroscopic treatment of OLT. Although the open modified Brostrom procedure has been proven effective for chronic lateral ankle instability, the benefits of minimal invasive arthroscopic procedure no longer exist when a lateral open incision is made.

More recently, an arthroscopic-assisted Brostrom technique has become increasingly popular [8]. Several comparative studies have demonstrated similar clinical results between the arthroscopic procedure and the open modified Brostrom procedure [15–19]. For patients accompanied by OLT, the arthroscopic-assisted Brostrom technique makes concurrent arthroscopic treatment possible. Nevertheless, there are few reports that compared the all-arthroscopic technique with the combined arthroscopic and open treatment in patients of lateral ankle instability accompanied by OLT [15–19]. In view of this, we aimed to test the following hypothesis in the present study: The outcome of all-arthroscopic surgery is no worse than that of the combined open and arthroscopic surgery for treating chronic lateral ankle instability accompanied by OLT.

## Methods

This retrospective study had been approved by the ethics committee of Xiangya Hospital of Central South University (China), and written consents had been acquired from all participants prior to implementation. The patients diagnosed of chronic lateral ankle instability and OLT, who underwent surgical treatment in the Department of Foot and Ankle Surgery in Xiangya Hospital from May 2015 to May 2017, were identified initially. The medical records of these patients were retrieved from the database of Xiangya Hospital and were examined for inclusion based on the following criteria: (1) recurrent instability of the ankle secondary to an injury to the lateral ligament complex and showing no response to conservative therapies including restriction of activities and administration of non-steroidal anti-inflammatory medicines for at least 3 months; (2) giving way; (3) persistent pain; and (4) OLT being detected on magnetic resonance imaging (MRI) images. The exclusion criteria include (1) history of previous surgery for lateral ankle instability, (2) history of previous fracture(s) of the affected ankle, (3) hyperlaxity, (4) neuromuscular disorder, (5) large OLT (with an area more than 150 mm^2^ or with an depth more than 8 mm on the MRI image), and (6) observation of syndesmosis widening in ankle arthroscopic evaluation.

Chronic lateral ankle instability is a condition characterized by a recurring giving way of the lateral side of the ankle. The anterior talofibular ligament (ATFL) was the weakest and the most commonly injured ligament among all three lateral ankle ligaments (i.e., ATFL, calcaneofibular ligament, and posterior talofibular ligament). The diagnosis of chronic lateral ankle instability was confirmed by the manual anterior drawer stress test (ADT) and the patient’s complaint of continuous ankle instability lasting for > 3 months. The ADT was performed with the lower leg hanging free and the knee maintaining flexed, while the ankle was positioned at 10°to 20°of plantar flexion. The tibia and the heel were stabilized by one hand each. The ADT was assessed bilaterally; a side-to-side difference above 5 mm in tibiotalar translation was regarded as positive [20].

**Fig. 1:**
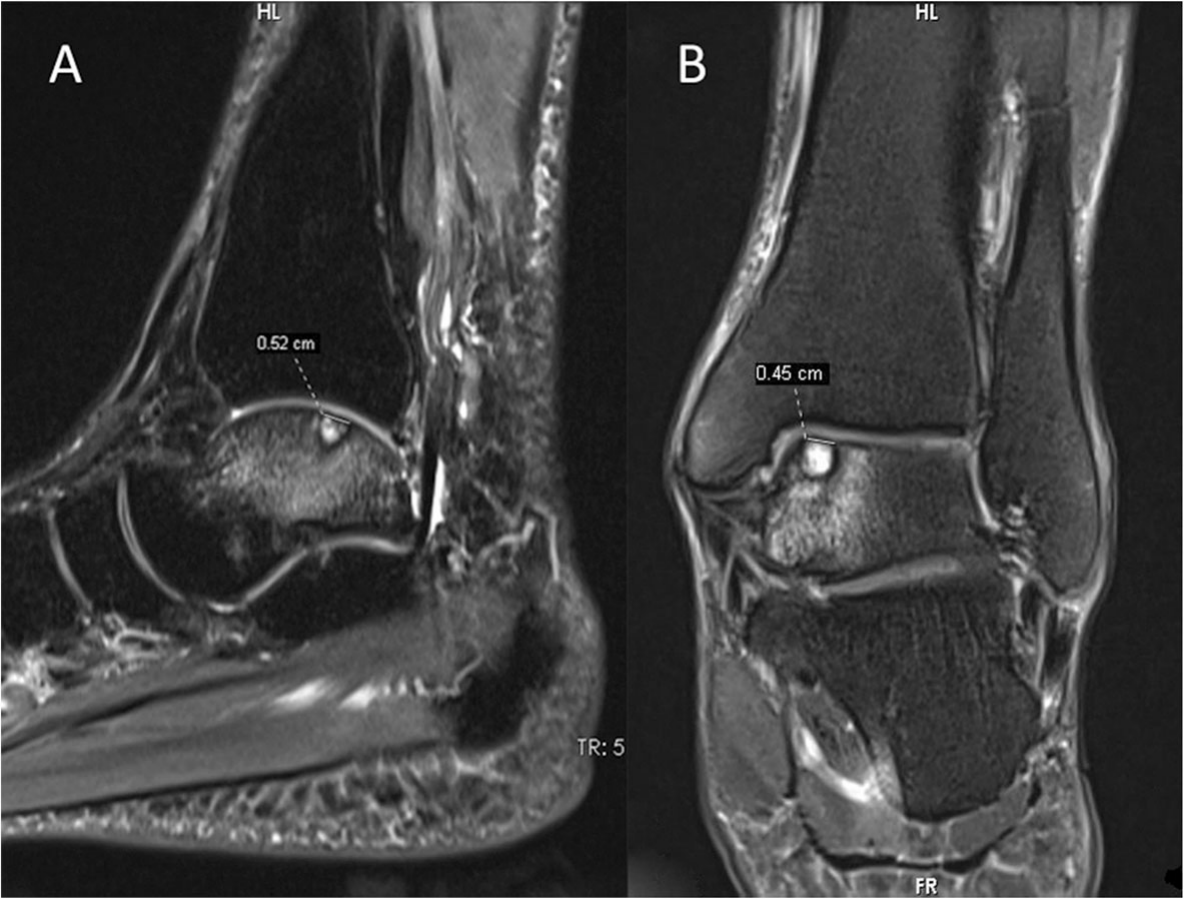
The diameter of OLT was measured on the sagittal (**a**) and coronal (**b**) imaging of MRI. The area of OLT was calculated by this equation: OLT area = coronal length × sagittal length × 0.79

OLTs were evaluated by MRI. All the patients had received MRI examination on the affected ankle using a 3.0-T MRI scanner before the operation (Fig. 1). During MRI examination, the ankle was placed in the neutral position, while coronal and sagittal imaging was performed using short time inversion recovery (STIR). The area of OLT was calculated by the following equation: OLT area = coronal length × sagittal length × 0.79 [21]. The patients with large OLTs who were required to undertake additional procedures rather than debridement or microfracture were excluded.

### Surgical technique

All the procedures were implemented in a standardized way by Liu Hua, a senior orthopedic surgeon with 10 years of working experience specialized in foot and ankle surgery. Initially, the patient was positioned in a supine pose under general anesthesia. With the inflation of the pneumatic tourniquet and noninvasive traction, a 2.7-mm, 30°arthroscope was used to ensure the best visualization and the lowest risk of iatrogenic injury to intra-articular structures. Standard anterolateral, anteromedial, and posterolateral portals were used. All the patients had received ankle arthroscopic evaluation of intra-articular pathology before performing lateral ankle stabilization. Choi et al. reported that some intra-articular lesions could result in unsatisfactory outcomes in lateral ligament reconstruction [22]. Therefore, the accompanying lesions in arthroscopic inspection were recorded carefully. Then, debridement (including soft tissue impingement, synovitis, ossicle, osteophyte, and loose body) was performed if necessary under arthroscopic observation.

**Fig. 2:**
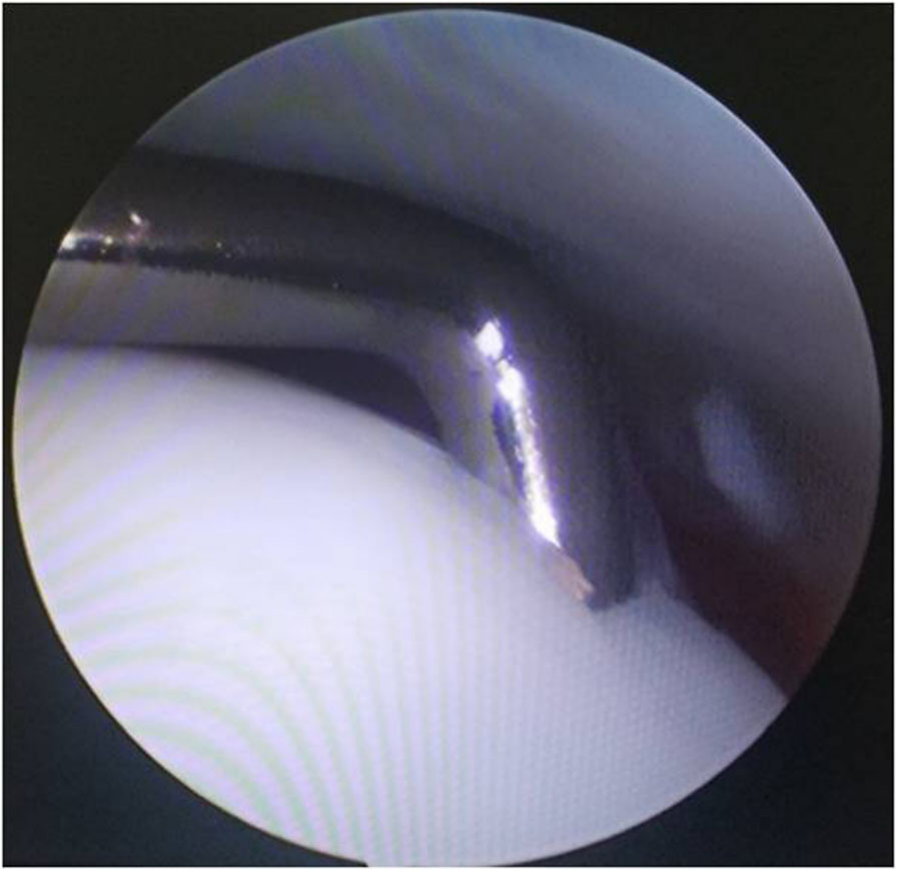
The OLT was carefully evaluated with a marked probe

When OLT was identified, the specific stage and size of the lesion would be carefully evaluated with a marked probe (Fig. 2). The OLTs can be classified into 6 stages in accordance with the Ferkel and Cheng [23] classification system as follows: stage A—smooth, intact, but soft or ballotable; stage B—rough surface; stage C—fibrillation/fissuring; stage D—presence of flap or bone exposure; stage E—loose, undisplaced fragment; and stage F—displaced fragment. For a cartilage with a rough surface or fibrillation (stage B-C), debridement would be implemented using a mechanical shaver (Dyonics Power Shaver System; Smith & Nephew, Andover, MA). For a lesion with a visible cartilage defect (stage D-F), microfracture would be performed alongside the debridement of the flapped cartilage and subchondral cyst in order to facilitate the revascularization of the lesion.

**Fig. 3:**
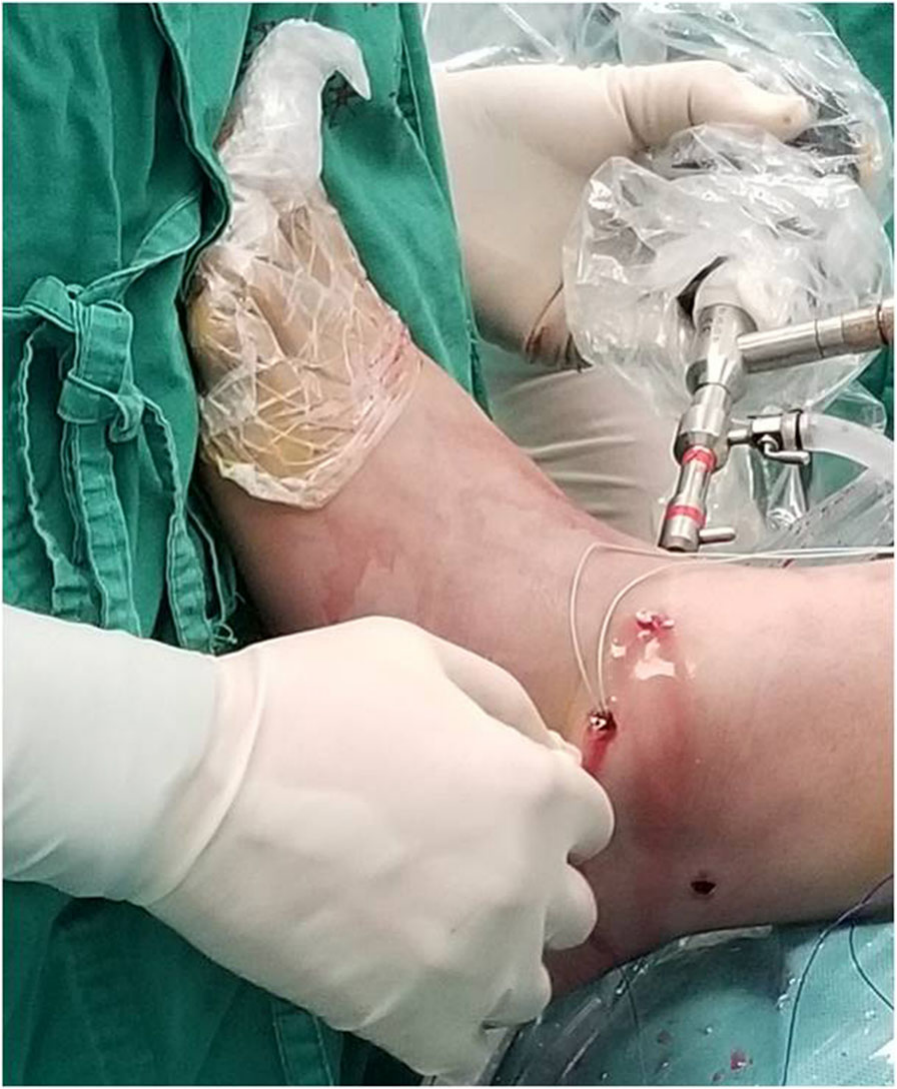
A 2.7-mm, 30°arthroscope was used for ankle arthroscopy. Standard anterolateral, anteromedial, and posterolateral portals were used for arthroscopic inspection. An accessory anterolateral (acAL) portal was created for arthroscopic-assisted Brostrom procedure

**Fig. 4:**
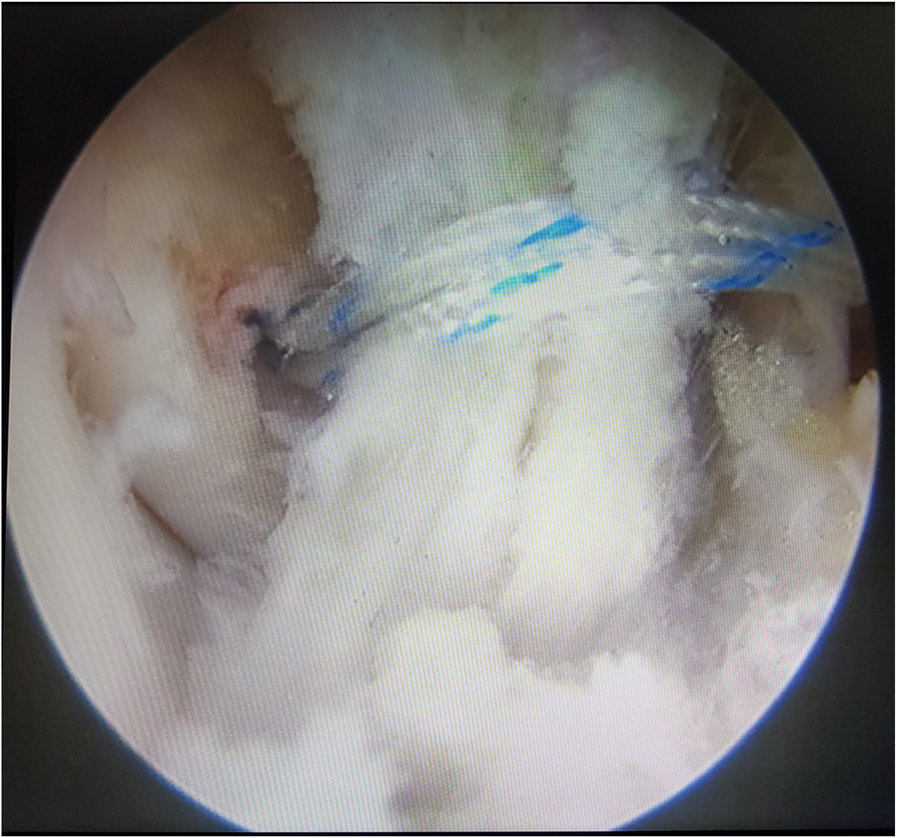
One suture anchors was inserted in the raw bone prepared in the fibula

**Fig. 5:**
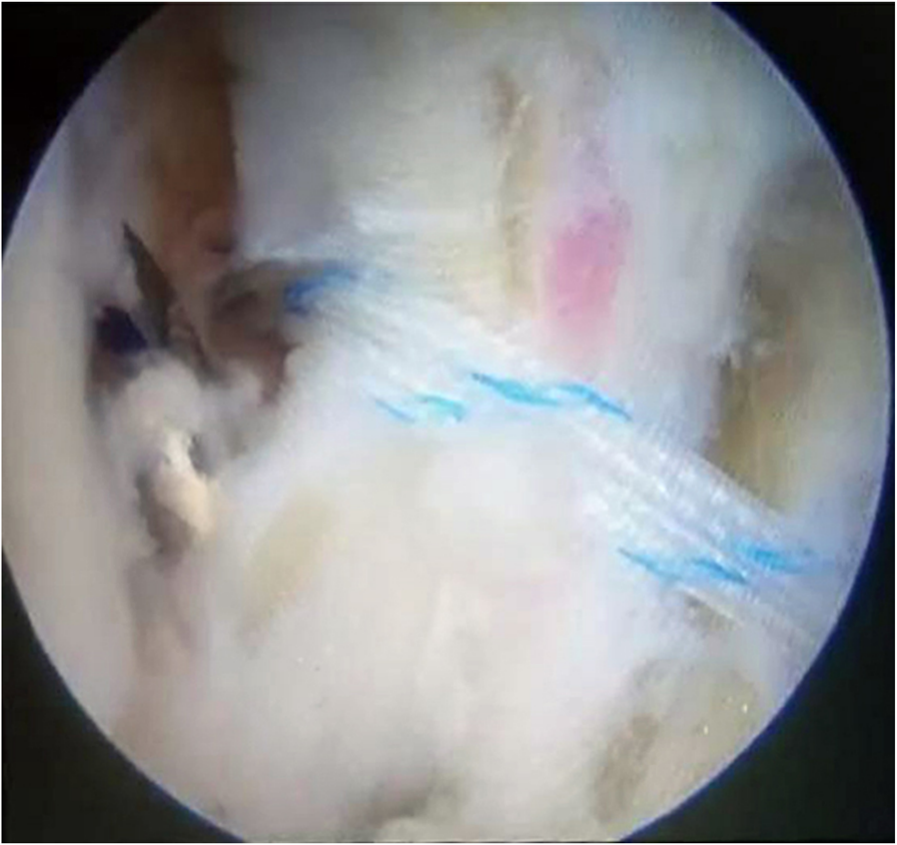
A tissue-penetrating instrument was used to penetrate the ATFL remnant. The blue PDS wire was located in the instrument

The arthroscopic-assisted Brostrom technique was performed following the same procedure described in a previous study [24]. To repair the ATFL, two initial anterior arthroscopic portals were utilized. The anterior lateral ankle joint was completely debrided to avoid potential postoperative impingement as far as possible, and the anterior distal face of the fibula was debrided subsequently with a shaver to create raw bone for promoting the adherence of soft tissues. Then, an accessory anterolateral (acAL) portal was created at the site 1.5 cm anterior to the tip of the fibula, passing through just proximal to the site of the raw bone prepared previously (Fig. 3). A drill/anchor guide was placed through the acAL portal and positioned in the raw bone prepared in the fibula, which was drilled for the purpose of inserting one or two suture anchors (2.8-mm, Arthrex, USA) inside (Fig. 4). Then, a tissue-penetrating instrument was used to penetrate the ATFL remnant (Fig. 5). Thereafter, the sutures were grasped to pass through the ATFL and then pulled cautiously to tighten the ATFL.

For the open modified Brostrom procedure, a curvilinear incision was created over the lateral malleolus. The ATFL remnant was identified and exposed, and one or two suture anchors were inserted into the fibula. The ATFL was tensioned under the eversion of the ankle and fixed to the fibula. Then, the proximal extensor retinaculum was exposed and moved from the attachment to the distal fibula to make sure that the repair of ATFL could be further reinforced.

### Postoperative rehabilitation

The postoperative rehabilitation protocol was developed under the guidance of a physical therapist. The affected ankle of the patient was immobilized by an ankle brace (A60; DJO Global) for 6 weeks. Isometric contraction of muscle groups around the ankle joint was allowed from the day after surgery. The passive and active range of motion (ROM) was allowed from the 7th day after surgery under the protection of ankle brace. The patient would then advance to partial weight bearing from the 5th week after surgery, and to full weight bearing from the 7th week after surgery over a period of 1 to 2 weeks. Generally, the patient was allowed to return to high-impact physical activities 6 months after surgery.

### Clinical evaluation methods

The patients were comprehensively examined both preoperatively and at 24 months postoperatively. Clinical evaluations, including subjective functional examination and ADT, were conducted by a professional orthopedic surgeon (Xu Can) based on the pain visual analog scale (VAS), the Karlsson ankle functional score (Karlsson score), the Tegner activity score, and the American Orthopaedic Foot & Ankle Society (AOFAS) score. A 10-point VAS (i.e., “0” indicates no pain and “10” indicates the highest level of imaginable pain) was used to evaluate the ankle pain during walking. A Karlsson scoring scale was formulated and used to evaluate the ankle function after the treatment for lateral ligament injuries of the ankle joint [25]. The Tegner activity scale is a numerical scale (0-10) with each value representing a specific activity [26]. The AOFAS ankle-hindfoot rating score is defined as a 100-point physician-based scale containing 3 components: function (45 points), pain (40 points), and alignment (15 points). All the patients were requested to grade their satisfaction on the overall treatment outcome at the last follow-up as “good,” “fair,” or “unsatisfactory;” however, they were not required to elaborate what influenced their specific sense of satisfaction.

### Statistical analysis

Although the present study was retrospective in nature, the calculation of sample size was conducted based on a non-inferiority design. The primary outcome used to determine the sample size was the Karlsson score at a minimum of 24-month follow-up. The 95% confidence interval (CI) was used to determine whether the postoperative difference in Karlsson score was within the pre-specified margin of non-inferiority, which was preset to be −6 points as suggested by previous studies [17]. Then, power analysis was carried out a priori to compute the minimum sample size required for detecting the 95% effect size at a 1-sided significance level (*P* < .05), and the results suggested that 30 patients were needed for each group in our study. The secondary outcomes include improvement in pain (VAS), the AOFAS score, the Tegner activity score, and the satisfaction rate. Meanwhile, the safety parameters including superficial peroneal nerve injuries and wound infection were also evaluated.

Statistical analyses were carried out with the SPSS software (Version 24.0 for Windows, IBM Corporation, Armonk, NY, USA). The continuous variables were expressed as mean ± standard deviation, while the categorical data were presented as counts and percentages. Wilcoxon’s signed-rank test was employed to compare the preoperative and postoperative functional outcome scores. Student’s *t* test, chi-square test, and Fisher’s exact test were performed to make necessary comparisons between the two groups. A 95% CI was used for analysis, and *P* < .05 was taken as statistically significant.

## Results

A total of 87 patients who qualified for the inclusion criteria and received surgical treatment by the same surgeon (Liu Hua) at Xiangya Hospital were included for analysis. Of these 87 patients, 8 were excluded because of large OLTs or syndesmosis widening, 5 withdrew after the surgery (2 arthroscopic and 3 open patients) and 7 were lost to follow-up (5 arthroscopic and 2 open patients). Eventually, 32 and 35 patients were included in the all-arthroscopic group and the open group, respectively. No significant differences in basic characteristics were observed between the two groups in terms of age, gender, body mass index, symptom duration, preoperative clinical score, and average follow-up period (Table 1).

**Table 1:** Preoperative data of demographics of the study groups

	All-arthroscopic group (*n* = 32)	Combined arthroscopic and open group (*n* = 35)	*P* value
Age, year	33.7 ± 7.0	35.8 ± 8.5	n.s
Gender, *n*	24males/8females	25males/10females	n.s
Body mass index, kg/m^2^	23.3 ± 2.3	24.1 ± 2.1	n.s
Symptom duration, month	30.1 ± 15.3	28.7 ± 13.7	n.s
Karlsson score	56.5 ± 11.5	59.3 ± 10.4	n.s
VAS score	8.0 ± 1.5	8.2 ± 1.5	n.s
AOFAS score	52.5 ± 11.5	53.3 ± 13.4	n.s
Tegner score	3(1-5)	3(1-5)	n.s
Average follow-up period, month	36.5 ± 12.7	39.1 ± 9.2	n.s

“n.s” stands for “no significant difference” between study groups

The intra-articular lesions are recorded in Table 2. As it can be seen, there were no significant differences between the two groups with respect to the accompanying abnormalities. The OLT size, location, and Ferkel stage are shown in Table 3. No significant differences were observed between the two groups.

**Table 2:** Comparison of accompanying abnormalities between the arthroscopic and open group

	All-arthroscopic group	Combined group	*P* value
Soft tissue impingement	25	24	n.s
Ossicle at lateral malleolus	4	3	n.s
Synovitis	6	6	n.s
Osteophyte	7	6	n.s
Loose body	6	5	n.s

“n.s” stands for “no significant difference” between study groups

**Table 3:** The OLT size, location, and Ferkel stage in terms of arthroscopic classification

	All-arthroscopic group	Combined group	*P* value
OLT size (on MRI images)			
Length	8.16 ± 3.3	9.2 ± 4.0	n.s
Width	5.0 ± 1.67	6.23 ± 1.72	n.s
Depth	4.7 ± 1.31	5.13 ± 1.4	n.s
Location			
Lateral	21	24	n.s
Medial	11	11	n.s
Ferkel and Cheng stage			
B-C	10	13	n.s
D-F	22	22	n.s

“n.s” stands for “no significant difference” between study groups

The ADT results of all the ankles in both groups were normal at the last follow-up. The Karlsson score, VAS score, Tegner score, and AOFAS score were significantly improved in both groups against the preoperative condition. All the patients managed to resume their pre-injury works after recovery from the surgery. Table 4 shows the statistical results of the Karlsson score, VAS score, Tegner score, AOFAS score, and satisfaction rate at the last follow-up for both groups. It can be seen that the clinical outcome at a minimum of 24-month follow-up was comparable between the two groups, and no significant difference in satisfaction rate was found.

**Table 4:** Comparison of clinical outcomes between the arthroscopic group and open group

	Mean ± SD	95% CI	*P* value
Karlsson score, mean ± SD			
All-arthroscopic group	83.1 ± 8.2	80.7, 85.3	0.89
Combined group	81.7 ± 9.1	79.9, 85.0	
VAS, mean ± SD			
All-arthroscopic group	1.8 ± 1.6	1.5, 2.2	0.73
Combined group	2.1 ± 1.7	1.7, 2.5	
AOFAS score, mean ± SD			
All-arthroscopic group	87.7 ± 7.6	86.0, 89.4	0.77
Combined group	86.9 ± 7.3	85.6, 88.7	
Tegner score			
All-arthroscopic group	5.5 ± 2.3(1-8)	5.0, 7.3	0.72
Combined group	5 ± 2.1(1-7)	4.5, 6.5	
Satisfaction rate, *n* (%)			
All-arthroscopic group	27(84.3%)	82.7%, 85.9%	0.69
Combined group	29(82.8%)	81.2%, 84.4%	

*P < 0.05 indicates statistical significance

Furthermore, 3 and 2 patients experienced superficial peroneal nerve injury in the all-arthroscopic group and the open group, respectively. Two patients suffered from knot pain in the all-arthroscopic group and 2 patients suffered from wound infection in the open group. No statistically-significant difference in the total complication rate was observed between the two groups.

## Discussions

Lateral ankle instability frequently coexists with intra-articular pathologic conditions, such as OLT, synovitis, impingement, osteophyte, and loose body. A part of lesions such as syndesmosis widening, OLT, and ossicles are regarded as significant predictors for the poor outcomes after lateral ankle reconstruction [22]. In this study, OLT was our primary research target, while other lesions were considered as confounding factors. In order to control the confounding bias, patients accompanied by syndesmosis widening were excluded. The lesions including soft tissue impingement, ossicle at lateral malleolus, synovitis, osteophyte, and loose body were recorded carefully, which however showed no significant differences between the two groups. The most important finding of our study is that the all-arthroscopic treatment of lateral ankle instability and OLT is no inferior to the open lateral ankle stabilization and arthroscopic treatment of OLT at a minimum of 24-month follow-up. As a matter of fact, the comparison suggested a slightly better result for arthroscopic surgery, but the difference was insignificant and did not support arthroscopic surgery as a preferred treatment.

Although the open modified Brostrom-Gould procedure is currently the gold standard for chronic lateral ankle instability, the arthroscopic repair of ATFL is being increasingly used in recent years. Several studies have compared the arthroscopic technique with the open repair technique. For example, Yeo et al. conducted a randomized controlled trial which showed no differences in AOFAS, VAS, and Karlsson scores between the all-inside arthroscopic and the open Brostrom operation at up to 1-year follow-up, [17]. Li et al. compared the AOFAS, Karlsson, and Tegner activity scores between the open and the arthroscopic lateral ankle repair, and concluded that these two procedures had similar outcomes at 2-year follow-up [15]. Matsui et al. compared the arthroscopic with the conventional open ATFL repair technique [16], and reported that the VAS and the Japanese Society for Surgery of the Foot Ankle-Hindfoot (JSSF) scale score showed no significant differences at 1-year follow-up. Rigby et al. performed a review of 62 patients who received either the open or the arthroscopic Brostrom procedure [18] and suggested no significant differences in the postoperative AOFAS, VAS, and Karlsson scores between the two techniques. Zeng et al. carried out a comparative study on the open and the arthroscopic ATFL repair techniques [19], which also suggested comparable therapeutic efficacy at 3-year follow-up. We noticed that the patients in Yeo, Li, and Rigby’ studies [15, 17, 18] involved several different accompanying intra-articular pathologies such as synovitis, anterior tibial spur, loose body, and OLTs. Matsui [16] and Zeng [19], however, enrolled only the patients without intra-articular lesions. To the authors’ best knowledge, our comparison is the first study that compared the results between the arthroscopic and the open surgery in patients of lateral ankle instability accompanied by OLTs.

Prior to the emergence of the arthroscopic technique, surgeons had to create an open exposure incision to accomplish lateral ankle stabilization when treating patients of lateral ankle instability accompanied by OLT. With arthroscopic ATFL repair, the Brostrom procedure can be performed directly after the arthroscopic treatment of OLT. Compared to the open surgery, the arthroscopic technique has a smaller skin incision and less subcutaneous dissection, which may consequently shorten the surgical time. Through the standard portal in the ankle arthroscopy, the blood vessels around the ATFL can also be well protected in order to facilitate the vascularization of the repaired ATFL [15]. Meanwhile, as a less invasive technique, the bleeding and postoperative adhesion of the arthroscopic technique in the ankle joint was lower than that of the open surgery. This can accelerate the process of recovery and postoperative rehabilitation [27], and therefore improve the short-term outcomes.

However, fast recovery mainly benefits those patients with only lateral ankle instability. In our study, the postoperative recovery seemed to be heavily influenced by the healing process of OLT. The patients who underwent only ATFL repair were encouraged to start ROM exercises and partial weight-bearing from the second day after surgery [16, 28], while for patients of OLT, the 3rd to the 8th week after surgery is regarded as the remodeling phase of OLT healing. In this period, the mesenchymal cells proliferate and differentiate into chondrocyte-like cells, which can produce a matrix containing type II collagens and proteoglycans [29]. To protect the formation of granulation and fibrocartilaginous tissue, most researchers suggested a period of 6 to 8 weeks of non-weight-bearing or partial weight-bearing [1, 30, 31]. Therefore, the fast recovery following a minimal invasive surgery did not yield much benefit for our patients. In order to improve the healing process of OLT, some reports also advocated early ROM exercises (to start within 1 week after surgery) and early weight-bearing (begin at 4 weeks after surgery) for OLT patients who received the treatment of microfracture [31]. In our study, passive and active ROM exercises under the protection of A60 ankle brace were allowed from the 7th day after surgery, while partial weight-bearing was allowed from the 5th week after surgery. This is a relatively aggressive rehabilitation protocol, but the non-weight-bearing period is still longer than that for patients with only lateral ankle instability.

The specific effect of OLT on the clinical outcome of chronic lateral ankle instability remained controversial. Choi and Hua [22, 32] evaluated the clinical outcomes of patients with chronic lateral ankle instability accompanied by intra-articular symptoms and reported that the modified Brostrom procedure coupled with ankle arthroscopy delivered satisfactory results, but the accompanying OLT was found to be associated with worse results. On the contrary, Li and Nery [20, 24] compared the outcomes of lateral ankle instability with and without OLT, and concluded that the functional outcomes of the two did not differ significantly from each other. We noticed that all the aforementioned studies did not report the detailed characteristics of OLT such as the location and lesion size, which however were widely accepted as prognostic factors when utilizing bone marrow stimulation for the surgical treatment of OLT [33]. The differences in clinical outcomes of these studies were probably attributed to the differences in the geometric features of OLT.

The minimal depth and area of OLT that lead to satisfactory outcomes are very important for determining which lesions should be treated alongside bone marrow stimulation. Historical guidelines suggested that the bone marrow stimulation for osteochondral lesion is < 150mm^2^ in area (or < 15 mm in diameter) [1, 21]. However, the latest study (published in 2018) recommended that the ideal size of lesion for bone marrow stimulation was < 10 mm in diameter, < 100mm^2^ in area, and < 5 mm in depth [33]. Generally speaking, smaller lesions correlate to better outcomes. Since our patients underwent the surgery between May 2015 and May 2017, we adopted the old guidelines. As shown in Table 3, the average lesion depth was 5.13 ± 1.4 mm and 4.7 ± 1.31 mm in the open and the all-arthroscopic group, respectively. The larger lesion depth in the open group may explain, at least in part, why the open group had slightly worse outcomes.

Our study has several limitations. First, as a retrospective study, no randomization was adopted. However, we observed neither significant differences in terms of the basic characteristics (see Table 2) nor significant differences in the lesion size, location, and Ferkel stage of OLT (Table 3) between the two groups. Second, the ADT was manually evaluated without taking stress radiographic images, which are considered more accurate for evaluating the stability of ankle. Third, MRI examination was not performed for evaluating OLT repair in the follow-up visits. Despite these limitations, the present study found no differences in terms of the efficacy and safety of outcomes between the all arthroscopic and the combined surgery for the treatment of chronic lateral ankle instability accompanied by OLT at a minimum of 24-month follow-up.

## Conclusions

In comparison with the open lateral ankle stabilization and arthroscopic treatment of OLT, the all-arthroscopic procedure showed no significant difference in clinical outcome at a minimum of 24-month follow-up. Despite the benefits of minimally invasive arthroscopic procedure combined with a relatively aggressive postoperative rehabilitation protocol, the clinical outcomes for patients with chronic lateral ankle instability accompanied by OLT did not yield significant improvement.

## Data Availability

The datasets referred to and/or analyzed in the present study can be retrieved from the corresponding author upon reasonable request.

## Abbreviations

OLT: Osteochondral lesion of talus
VAS: Visual analog scale
AOFAS: American Orthopaedic Foot & Ankle Society Score
MRI: Magnetic resonance imaging
ADT: Anterior drawer stress test
ROM: Range of motion
ATFL: Anterior talofibular ligament
JSSF: Japanese Society for Surgery of the Foot Ankle-Hindfoot
OATS: Osteochondral autograft transfer systems
STIR: Short time inversion recovery
CI: Confidence interval

## References

[1] Chuckpaiwong B, Berkson EM, Theodore GH (2008). Microfracture for osteochondral lesions of the ankle: outcome analysis and outcome predictors of 105 cases. Arthroscopy.

[2] Hintermann B, Boss A, Schafer D (2002). Arthroscopic findings in patients with chronic ankle instability. Am J Sports Med.

[3] Komenda GA, Ferkel RD (1999). Arthroscopic findings associated with the unstable ankle. Foot Ankle Int.

[4] Sugimoto K, Takakura Y, Okahashi K, Samoto N, Kawate K, Iwai M (2009). Chondral injuries of the ankle with recurrent lateral instability: an arthroscopic study. J Bone Joint Surg Am..

[5] Takao M, Uchio Y, Naito K, Fukazawa I, Ochi M (2005). Arthroscopic assessment for intra-articular disorders in residual ankle disability after sprain. Am J Sports Med.

[6] Harrington KD (1979). Degenerative arthritis of the ankle secondary to long-standing lateral ligament instability. J Bone Joint Surg Am..

[7] Georgiannos D, Bisbinas I, Badekas A (2016). Osteochondral transplantation of autologous graft for the treatment of osteochondral lesions of talus: 5- to 7-year follow-up. Knee Surg Sports Traumatol Arthrosc..

[8] Yasui Y, Murawski CD, Wollstein A, Takao M, Kennedy JG. Operative treatment of lateral ankle instability. JBJS Rev. 2016;4:e6–6. 10.2106/JBJS.RVW.15.00074 27490220

[9] Yasui Y, Takao M, Miyamoto W, Matsushita T (2014). Simultaneous surgery for chronic lateral ankle instability accompanied by only subchondral bone lesion of talus. Arch Orthop Trauma Surg..

[10] Polat G, Erşen A, Erdil ME, Kızılkurt T, Kılıçoğlu Ö, Aşık M (2016). Long-term results of microfracture in the treatment of talus osteochondral lesions. Knee Surg Sports Traumatol Arthrosc..

[11] Takao M, Komatsu F, Naito K, Uchio Y, Ochi M (2006). Reconstruction of lateral ligament with arthroscopic drilling for treatment of early-stage osteoarthritis in unstable ankles. Arthroscopy.

[12] Niemeyer P, Salzmann G, Schmal H, Mayr H, Südkamp NP (2012). Autologous chondrocyte implantation for the treatment of chondral and osteochondral defects of the talus: a meta-analysis of available evidence. Knee Surg Sports Traumatol Arthrosc.

[13] Gregush RV, Ferkel RD (2010). Treatment of the unstable ankle with an osteochondral lesion: results and long-term follow-up. Am J Sports Med.

[14] Jiang D, Ao YF, Jiao C, Xie X, Chen LX, Guo QW, Hu YL (2018). Concurrent arthroscopic osteochondral lesion treatment and lateral ankle ligament repair has no substantial effect on the outcome of chronic lateral ankle instability. Knee Surg Sports Traumatol Arthrosc..

[15] Li H, Hua Y, Li H, Ma K, Li S, Chen S (2017). Activity level and function 2 years after anterior talofibular ligament repair: a comparison between arthroscopic repair and open repair procedures. Am J Sports Med.

[16] Matsui K, Takao M, Miyamoto W, Matsushita T (2016). Early recovery after arthroscopic repair compared to open repair of the anterior talofibular ligament for lateral instability of the ankle. Arch Orthop Trauma Surg..

[17] Yeo ED, Lee KT, Sung IH, Lee SG, Lee YK (2016). Comparison of all-inside arthroscopic and open techniques for the modified Brostrom procedure for ankle instability. Foot Ankle Int.

[18] Rigby RB, Cottom JM (2019). A comparison of the “All-Inside” arthroscopic Brostrom procedure with the traditional open modified Brostrom-Gould technique: a review of 62 patients. Foot Ankle Surg..

[19] Zeng G, Hu X, Liu W, Qiu X, Yang T, Li C, Song W (2020). Open Brostrom-Gould repair vs arthroscopic anatomical repair of the anterior talofibular ligament for chronic lateral ankle instability. Foot Ankle Int.

[20] Li H, Hua Y, Li H, Li S, Ma K, Chen S (2018). Treatment of talus osteochondral defects in chronic lateral unstable ankles: small-sized lateral chondral lesions had good clinical outcomes. Knee Surg Sports Traumatol Arthrosc..

[21] Choi WJ, Park KK, Kim BS, Lee JW (2009). Osteochondral lesion of the talus: is there a critical defect size for poor outcome?. The American journal of sports medicine.

[22] Choi WJ, Lee JW, Han SH, Kim BS, Lee SK (2008). Chronic lateral ankle instability: the effect of intra-articular lesions on clinical outcome. Am J Sports Med.

[23] Ferkel RD, Zanotti RM, Komenda GA, Sgaglione NA, Cheng MS, Applegate GR, Dopirak RM (2008). Arthroscopic treatment of chronic osteochondral lesions of the talus: long-term results. Am J Sports Med.

[24] Nery C, Raduan F, Del Buono A, Asaumi ID, Cohen M, Maffulli N (2011). Arthroscopic-assisted Brostrom-Gould for chronic ankle instability: a long-term follow-up. Am J Sports Med.

[25] Karlsson J, Peterson L (1991). Evaluation of ankle joint function: the use of a scoring scale. Foot..

[26] Tegner Y (1985). Rating systems in the evaluation of knee ligament injuries. Clin Orthop Relat Res..

[27] Matsui K, Burgesson B, Takao M, Stone J, Guillo S, Glazebrook M (2016). Minimally invasive surgical treatment for chronic ankle instability: a systematic review. Knee Surg Sports Traumatol Arthrosc.

[28] Song Y, Li H, Sun C, Zhang J, Gui J, Guo Q, Song W, Duan X, Wang X, Wang X (2019). Clinical guidelines for the surgical management of chronic lateral ankle instability: a consensus reached by systematic review of the available data. Orthop J Sports Med.

[29] van Eekeren IC, Reilingh ML, van Dijk CN (2012). Rehabilitation and return-to-sports activity after debridement and bone marrow stimulation of osteochondral talar defects. Sports Med.

[30] Saxena A, Eakin C (2007). Articular talar injuries in athletes: results of microfracture and autogenous bone graft. Am J Sports Med.

[31] D’Hooghe P, Murawski CD, Boakye LAT, Osei-Hwedieh DO, Drakos MC, Hertel J, Lee KB, Popchak A, Wiewiorski M, van Dijk CN (2018). Rehabilitation and return to sports: proceedings of the International Consensus Meeting on Cartilage Repair of the Ankle. Foot Ankle Int.

[32] Hua Y, Chen S, Li Y, Chen J, Li H (2010). Combination of modified Brostrom procedure with ankle arthroscopy for chronic ankle instability accompanied by intra-articular symptoms. Arthroscopy.

[33] Hannon CP, Bayer S, Murawski CD, Canata GL, Haverkamp D, Lee JW, O’Malley MJ, Yinghui H, Stone JW, Clanton TO (2018). Debridement, curettage, and bone marrow stimulation: proceedings of the International Consensus Meeting on Cartilage Repair of the Ankle. Foot Ankle Int.

